# Mangiferin Relieves Lipopolysaccharide-Induced Injury by Up-Regulating miR-181a *via* Targeting PTEN in ATDC5 Cells

**DOI:** 10.3389/fphar.2020.00137

**Published:** 2020-03-05

**Authors:** Yunfei Ma, Ying Liu, Yunyan Ma, Nan Jiang, Lei Wang, Bowei Wang, Wanting Niu, Yanjun Hu, Qingrong Lin, Bin Yu

**Affiliations:** ^1^ Department of Orthopaedics and Traumatology, Nanfang Hospital, Southern Medical University, Guangzhou, China; ^2^ Department of Orthopaedic Surgery, Binzhou Medical University Hospital, Binzhou, China; ^3^ Department of Obstetrics, Guangdong Second Provincial General Hospital, Guangzhou, China; ^4^ Tissue Engineering Laboratories, VA Boston Healthcare System, Boston, MA, United States; ^5^ Department of Orthopedics, Brigham and Women’s Hospital, Harvard Medical School, Boston, MA, United States

**Keywords:** osteoarthritis, mangiferin, PTEN, miR-181a, NF-κB, PTEN/PI3K/AKT pathways

## Abstract

**Background:**

Mangiferin (MF) was reported to possess anti-inflammatory activity. This investigation tried to probe into the underlying mechanism of MF in osteoarthritis.

**Methods:**

ATDC5 cells were pretreated with series concentrations of MF (0.1, 1, 5, 10, 15, 20 μM) for 2 h and then were exposed to lipopolysaccharide (LPS) (5 μg/ml) for 12 h to construct the inflammatory injury model. The cell viability, productions of pro-inflammatory cytokines and enzymes were respectively measured by employing CCK-8 assay, western blot, ELISA, and quantitative reverse-transcription (qRT)-PCR. miR-181a expression was altered by employing cell transfection. Dichloro-dihydro-fluorescein diacetate (DCFH-DA) method was employed for detection of reactive oxygen species (ROS) generation. Dual luciferase activity assay was conducted for analyzing the relationship between miR-181a and PTEN. The underlying mechanism was determined by employing western blot.

**Results:**

High doses of MF treatment (15 and 20 μM) noticeably induced inflammatory injury exhibiting as increased the productions of pro-inflammatory cytokines, enzymes and ROS, activated NF-κB pathway and deactivated PTEN/PI3K/AKT pathway in ATDC5 cells. Besides, MF treatment notably remitted LPS-induced inflammatory injury through deactivation of NF-κB pathway and activation of PTEN/PI3K/AKT pathway. PTEN was a target of miR-181a. Inhibition of miR-181a remarkably reversed MF-triggered impacts on ATDC5 cells.

**Conclusion:**

MF attenuated LPS-induced inflammatory damage through miR-181a/PTEN axis and thereby inhibiting NF-κB pathway and activating PI3K/AKT pathway.

## Introduction

Osteoarthritis (OA) is the commonest osteoarticular disease and one of the vital reasons for physical diseases in the elderly (Ameye and Chee, 2006). Symptoms mainly include joint pain during activity, joint stiffness after transient inactivity, and joint cracking (Dieppe and Lohmander, 2005). Among which, joint pain is deemed to be the especially important, and OA is recognized as the primary cause of high rate of joint pain in the elderly (Peat et al., 2001). To date, although growing numbers of investigations have tried to explore effective therapeutic strategies for OA (Bellamy et al., 2006a; Bellamy et al., 2006b; Elron-Gross et al., 2009; Homma et al., 2009), there are still defects, such as side effects, so seeking for new drugs that used for OA treatment is still in urgent need.

Mangiferin (MF) is the most widely studied plant polyphenolic compound, which is ubiquitous in *Mangifera indica* and *Salacia reticulata* and possesses various pharmacological activities, including anti-neoplastic (Noratto et al., 2010; Garcia-Rivera et al., 2011), anti-inflammatory (Garrido et al., 2004; Mohan et al., 2013), antioxidant (Ribeiro et al., 2008; Pourahmad et al., 2010), and immunomodulatory (Makare et al., 2001) activities. Studies have already been conducted to investigate the influences of MF on bone-related diseases. For example, MF was reported to be effective in the treatment and prevention of mixed osteoarthritic pain (Garrido-Suarez and Garrido, 2019). Besides, an earlier literature clarified that MF exhibited anti-osteoclastogenic effects in the prevention and treatment of bone diseases, such as osteoporosis, erosive arthritis, and so on (Ang et al., 2011). Moreover, it was disclosed that MF played its protective roles in osteoarthritic chondrocytes through inhibiting *tert*-butyl hydroperoxide (TBHP)-triggered extracellular matrix (ECM) degradation and apoptosis (Li et al., 2019). In addition to this, another study reported that MF ameliorated interleukin (IL)-1β-triggered inhibitory effects on chondrogenic differentiation of mesenchymal stem cells in OA (Huh et al., 2014). Whereas, the underlying mechanisms by which MF functions in the treatment of OA still remain unclear.

A previous research once reported that there were several microRNAs (miRNAs) participated in the processes of OA pathogenesis (Goldring and Marcu, 2012). miR-181a was one of the miRNAs that was mentioned to be associated with OA pathogenesis (Okuhara et al., 2012). Additionally, an earlier research specified that miR-181a-5p took part in the modulation of cell activities in facet joints chondrocytes (Nakamura et al., 2016). Moreover, another research specified that miR-181a expression was decreased in OA while increased in the synovial fluid which was derived from hyaluronic acid treated OA patients (Xu et al., 2015b). Nevertheless, whether miR-181a is involved in the regulation of MF in OA still remains unknown.

This research made an attempt to investigate the function and potential mechanism of MF on lipopolysaccharide (LPS)-evoked ATDC5 cells. ATDC5 cells are derived from mouse teratoma cancer cell line AT805. As a pre-chondrocyte cell line, the differentiation process of ATDC5 cells is similar to that of cartilage formation. Besides, ATDC5 cells have been widely used as a research model of OA *in vitro* (Wu et al., 2017a; Jin et al., 2018; Liu et al., 2019). Therefore, we performed LPS treatment on ATDC5 cells to construct the research model in this investigation and the results showed that MF treatment notably remitted LPS-induced inflammatory injury, deactivated nuclear factor kappa-B (NK-κB) pathway and activated phosphatidylinositol 3 kinase/protein kinase B (PI3K/AKT) pathway. Moreover, following experiment disclosed that PTEN was a target of miR-181a and miR-181a inhibition noticeably reversed MF-triggered impacts on LPS-induced ATDC5 cells. This investigation might contribute to the discovery of new therapeutic drugs and targets for OA treatment.

## Methods and Materials

### Cell Culture and Treatment

This research was approved by the ethics committee of Nanfang Hospital, Southern Medical University (Guangzhou, China). The ATDC5 cells used in this study were attained from the American Type Culture Collection (ATCC, Rockville, MD, USA). The ATDC5 cells were sustained in a culture medium comprising 90% Dulbecco's Modified Eagle's Medium/F12 (DMEM/F12, BBI Solution, Crumlin, UK) and 10% fetal bovine serum (FBS, BBI Solution) under 5% CO_2_ and 37°C condition. When the confluency reached 90%, ATDC5 cells were trypsinized with 0.25% trypsin/ethylenediaminetetraacetic acid (EDTA) (Thermo Fisher Scientific, Grand Island, USA) and then were plated in six-well plates. Subsequent experiments were conducted after the cells attached for 8 h.

ATDC5 cells were treated with LPS (5 μg/ml) (No. L2630-25MG; Sigma, St. Louis, USA) (serotype: *Escherichia coli* O111:B4; EINECS: 297-473-0; MDL number: MFCD00164401; NACRES: NA. 25) for 12 h to construct the inflammatory injury model. Moreover, ATDC5 cells were dealt with series concentrations (0.1, 1, 5, 10, 15, 20 μM) of MF (Sigma) and precultured in an incubator at 37°C for 2 h before LPS inducement. Additionally, ATDC5 cells were pretreated with 10 μM of NAC [N-acetylcysteine, a scavenger of reactive oxygen species (ROS)] (Sigma) at 37°C for 1 h before LPS inducement to serve as the positive control of MF treatment (Xu et al., 2015a).

Additionally, for investigation of the signal pathways, the LPS + MF treated ATDC5 cells were respectively incubated with the PI3K inhibitor Wortmannin (MedChemExpress, New Jersey, USA) (10 μM, 1 h), PTEN inhibitor VO-OHpic trihydrate (MedChemExpress) (10 nM, 1 h), AKT inhibitor MK2206 (MedChemExpress) (200 nM, 30 min) and NF-κB pathway inhibitor pyrrolidine dithiocarbamate (PDTC) (Sigma) (10 μM, 30 min) reference to earlier published literatures (Xu et al., 2015a; Lu et al., 2017; Guo et al., 2018; Masarwi et al., 2018).

### Cell Counting Kit-8 (CCK-8) Assay

After transfection and treatment, cell viability was measured by using CCK-8 assay (Dojindo, Tokyo, Japan). ATDC5 cells were plated in 96-well plates (5 × 10^3^ cells per well) and maintained in an incubator under 5% CO_2_ and 37°C condition. After the cells were attached, 0.1, 1, 5, 10, 15, and 20 μM of MF were respectively provided and the mixtures were maintained in the same incubator for 2 h. Another group was firstly exposed to appropriate concentration of MF for 2 h, and then induced with LPS for 12 h. Afterwards, 10 μl of CCK-8 reagent was supplied to each well and the mixtures were maintained in the incubator for 1 h at 37°C. Finally, the absorbance at 450 nm was detected by utilizing a microplate reader (PerkinElmer, Fremont, CA, USA). The cell viabilities of each group were calculated according to the kit instructions.

### Western Blot

After MF + LPS treatment was finished for 60 min, the cytoplasmic and nuclear proteins were respectively extracted by utilizing NE-PER™ Nuclear and Cytoplasmic Extraction Reagents (Thermo Fisher Scientific) following the product instructions. The concentrations of these proteins were tested by using the BCA method (Thermo Fisher Scientific). The proteins were separated by sodium dodecyl sulfate polyacrylamide gel electrophoresis (SDS-PAGE), and then were transferred onto the polyvinylidene difluoride (PVDF) membranes. After that, the PVDF membranes were blocked in 5% bovine serum albumin (BSA; Thermo Fisher Scientific) at 25°C for 2 h, and then were maintained with primary antibodies directly against interleukin (IL)-1β (ab9722, Abcam, Cambridge, UK), IL-6 (ab208113, Abcam), tumor necrosis factor (TNF)-α (ab34674, Abcam), inducible nitric oxide synthase (iNOS) (ab15323, Abcam), cyclooxygenase-2 (Cox-2) (ab52237, Abcam), PTEN (residue Ser380) (ab31392, Abcam), NK-κB-p65 (#8242, Cell Signaling Technology, Inc., MA, USA), t-IκBα (9242, Cell Signaling Technology, Massachusetts, USA), p-IκBα (2859, Cell Signaling Technology), t-PI3K (ab227204, Abcam), p-PI3K (ab182651, Abcam), t-AKT (ab64148, Abcam), p-AKT (ab133458, Abcam), H3 (ab61251, Abcam), α-tubulin (ab24246, Abcam), and β-actin (ab227387, Abcam) at 4°C overnight. Subsequently, the PVDF membranes were rinsed with Tris-buffered saline tween (TBST) for three times, and then were maintained with HRP-labeled secondary antibody (ab205718, Abcam) for 1.5 h. After rinsed again, color development was performed by utilizing a DAB kit (Thermo Fisher Scientific) and the gray value of each protein band was obtained by using Image Lab software (Bio-Rad, California, USA) analysis.

### Enzyme-Linked Immunosorbent Assay (ELISA)

After MF + LPS treatment was finished for 60 min, the levels of pro-inflammatory cytokines IL-6, IL-1β and TNF-α in the collected supernatant were respectively examined by utilizing mouse IL-1β ELISA Kit (ab100704, Abcam), mouse IL-6 ELISA Kit (ab100712, Abcam), and mouse TNF-α ELISA Kit (ab208348, Abcam) according to the product manual. The standard solutions were respectively prepared by using the reagents provided in the kits. The absorbance values of each well were collected by using a microplate reader (PerkinElmer, Fremont, CA, USA). Data analysis was conducted in SPSS18.0 software (SPSS, Inc., Chicago, USA).

### Intracellular Reactive Oxygen Species (ROS) Assay

After MF + LPS treatment was finished for 30 min, the ATDC5 cells were collected and maintained with the dichloro-dihydro-fluorescein diacetate (DCFH-DA) probe solution (final concentration 10 μmol/l; Thermo Fisher Scientific) for 30 min. Subsequently, the probe incubation solution was discarded. Cells were washed with Dulbecco's phosphate buffered saline (DPBS) for three times, and centrifuged at 1,000 × g for 5 min. Afterwards, cells were suspended in 500 μl of DPBS and the results were analyzed on the flow cytometry (Beckman Coulter, USA).

### Cell Transfection

The sequences of miR-181a inhibitor and negative control (NC) inhibitor were both synthesized by Thermo Fisher Scientific (Grand Island, USA). These sequences were respectively transfected into ATDC5 cells by utilizing Lipofectamine 3000 (Invitrogen) according to the product instructions. Cell collection was performed 48 h post transfection and the transfection efficiency was detected by using quantitative reverse-transcription polymerase chain reaction (qRT-PCR). The sequences used in transfection were detailed as below: miR-181a inhibitor: 5’-ACUCACCGACAGCGUUGAAUGUU-3’; NC inhibitor: 5’-CGACCGAACCGGAAGGCCCACCU-3’.

### Dual Luciferase Activity Assay

The wild type (wt) and mutant type (mut) of mouse PTEN promoter fragment (1,600 base pair, upstream from the start codon ATG) was both cloned from the mouse genome and respectively constructed into the luciferase reporter pMiR to generate pMiR-report vector (PTEN-wt and PTEN-mut). Afterwards, the recombinant vectors were respectively transformed into *E. coli* DH5α competent cells (Thermo Fisher Scientific) for further amplification. Then, the cells were co-transfected with PTEN-mut/PTEN-wt and miR-181a mimic/NC mimic by utilizing Lipofectamine 3000 (Invitrogen). The relative activity of luciferase was measured by employing a dual-luciferase reporter assay system (Promega, Madison, Wisconsin, USA) following the procedures and instructions.

### qRT-PCR

Total cellular miRNA and messenger RNA (mRNA) was isolated by utilizing TRIzol and their concentrations were examined by the use of the Nanodrop 2000 system (Thermo Fisher Scientific). Complementary DNA (cDNA) was synthesized from 1 μg of total RNA by utilizing the PrimeScript RT reagent kit (Thermo Fisher Scientific). The expression levels of IL-6, IL-1β, TNF-α, iNOS, Cox-2 and miR-181a were detected by the use of the Fast SYBR Green Master Mix (Thermo Fisher Scientific) on a Bio-Rad CFX 96 real time detection system (Bio-Rad, California, USA). Results were analyzed by using the CFX Manager software (Bio-Rad). The reaction conditions were as follows: initial degeneration (94°C) for 5 min, 30 cycles of degeneration (94°C) for 50 s, annealing (55°C) for 50 s, and extension (72°C) for 1 min, and then final extension (72°C) for 10 min. β-actin and U6 were respectively used as the internal reference for mRNAs and miRNA. Relative expression of these genes was respectively calculated by using 2^–ΔΔCT^ method. The primers used in this study were listed in **Table 1**.

**Table 1:** Primer sequences used in the quantitative reverse transcription (qRT)-PCR analysis.

Gene name	Sequences
IL-6	Forward: 5'-CTGCAAGAGACTTCCATCCAG-3'
	Reverse: 5'-AGTGGTATAGACAGGTCTGTTGG-3'
IL-8	Forward: 5'-ACCACACTGCGCCAACACAGAAAT-3'
	Reverse: 5'-TCCAGACAGAGCTCTCTTCCATCAGA-3'
TNF-α	Forward: 5'-CAGGGCAATGATCCCAAAGTA-3'
	Reverse: 5'-GCAGTCAGATCATCTTCTCGA-3'
iNOS	Forward: 5'-CACCTTGGAGTTCACCCAGT-3'
	Reverse: 5'-ACCACTCGTACTTGGGATGC-3'
Cox-2	Forward: 5'-TCCATTGACCAGAGCAGAGA-3'
	Reverse: 5'-TCTGGACGAGGTTTTTCCAC-3'
β-actin	Forward: 5'-CGTGCGTGACATCAAAGAGAA-3'
	Reverse: 5'-TGGATGCCACAGGATTCCCAT-3'
miR-181a	Forward: 5'-CGCAACATTCAACGCTGTC -3'
	Reverse: 5'-GTGCAGGGTCCGAGGT-3'
U6	Forward: 5'-TGGGGTTATACATTGTGAGAGGA-3'
	Reverse: 5'-GTGTGCTACGGAGTTCAGAGGTT-3'

### Statistical Analysis

Each experiment had three replications. Data were presented as the mean ± standard deviation (SD). Data were analyzed by utilizing SPSS 18.0 software (SPSS, Inc., Chicago, USA). Comparison among multi-groups was performed by using One-way analysis of variance (ANOVA), and comparison between two groups was conducted by using Student’s t test. *P* < 0.05 was acknowledged as statistically significant.

## Result

### High Concentrations of MF Triggered Inflammatory Injury in ATDC5 Cells

**Figure 1:**
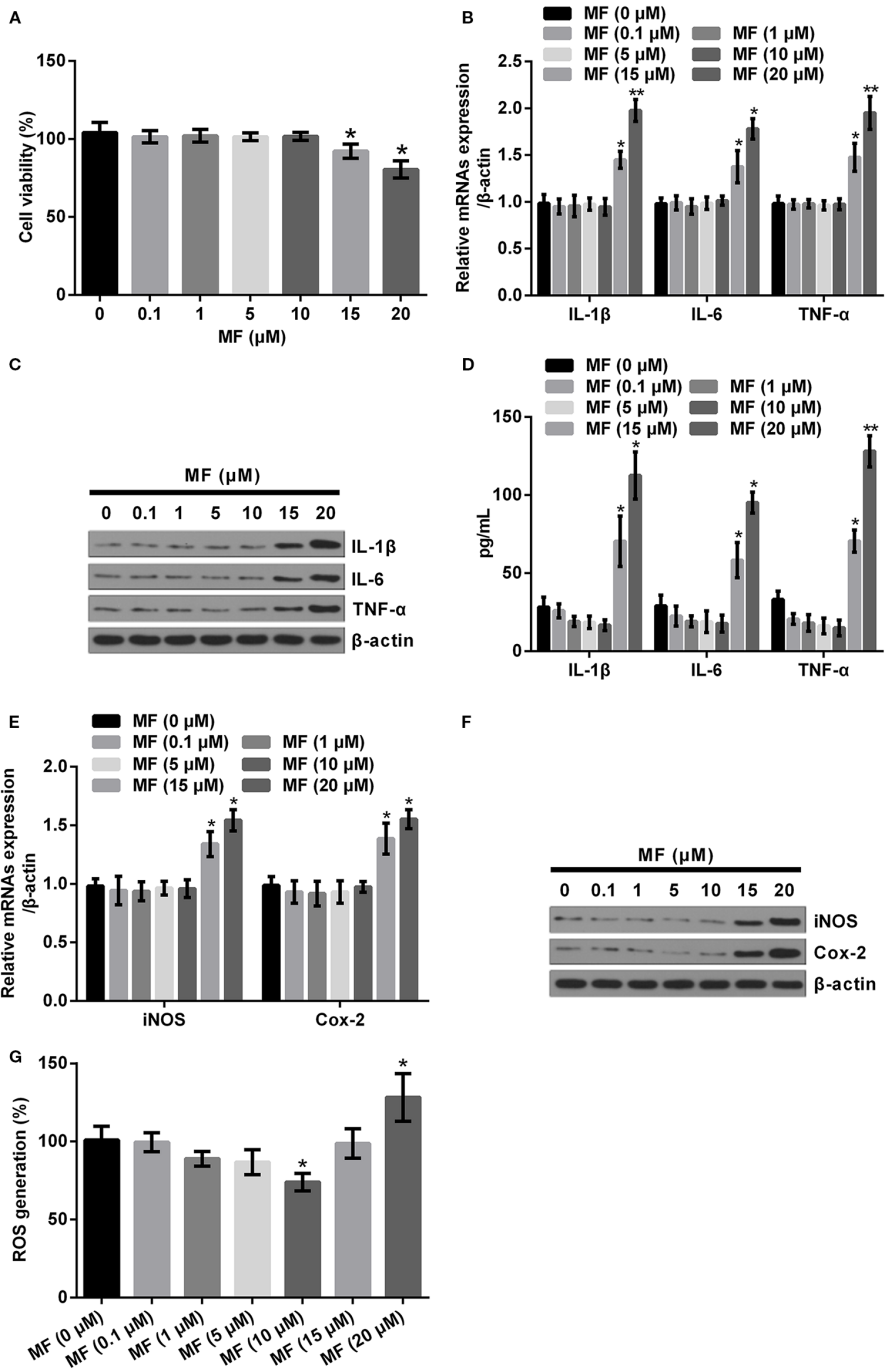
High doses of MF treatment triggered inflammatory injury in ATDC5 cells. ATDC5 cells were exposed to series concentrations of MF (0.1, 1, 5, 10, 15, and 20 μM). **(A)** 15 and 20 μM of MF treatment remarkably decreased the viability of ATDC5 cells. **(B**–**D)** 15 and 20 μM of MF treatment noticeably increased the mRNA expression and protein productions of pro-inflammatory cytokines [interleukin (IL)-1β, IL-6, and TNF-α]. **(E**, **F)** 15 and 20 μM of MF treatment markedly enhanced the messenger RNA (mRNA) expression and protein productions of pro-inflammatory enzymes (iNOS and Cox-2). **(G)** 10 μM of MF treatment notably declined ROS generation, while 20 μM of MF treatment markedly increased ROS generation. MF, mangiferin; IL, interleukin; TNF-α, tumor necrosis factor-α; iNOS, inducible nitric oxide synthase; Cox-2, cyclooxygenase-2; ROS, reactive oxygen species. *P < 0.05, **P < 0.01.

For determination of the influences of MF treatment on ATDC5 cells, series concentrations of MF treatment were conducted on ATDC5 cells. Results showed that low concentrations of MF treatment (0.1–10 μM) had no significant influences on the viability and productions of pro-inflammatory cytokines and enzymes. However, results also displayed that when the concentration increased to 15 and 20 μM, the cell viability was markedly decreased, while the mRNA expression and protein productions of pro-inflammatory cytokines and enzymes were all notably increased compared with control (*P* < 0.05 or *P* < 0.01, **Figures 1A–F**). Moreover, detection of ROS generation also demonstrated that 10 μM of MF treatment remarkably decreased ROS generation (*P* < 0.05), while 20 μM of MF treatment markedly increased ROS generation (*P* < 0.05, **Figure 1G**). These observations indicated that MF treatment might perform protective efficacy on ATDC cells at certain concentrations, however, excessive concentrations of MF could trigger opposite effects.

### High Concentrations of MF Activated NF-κB Pathway, While Deactivated PTEN/PI3K/AKT Pathway

**Figure 2:**
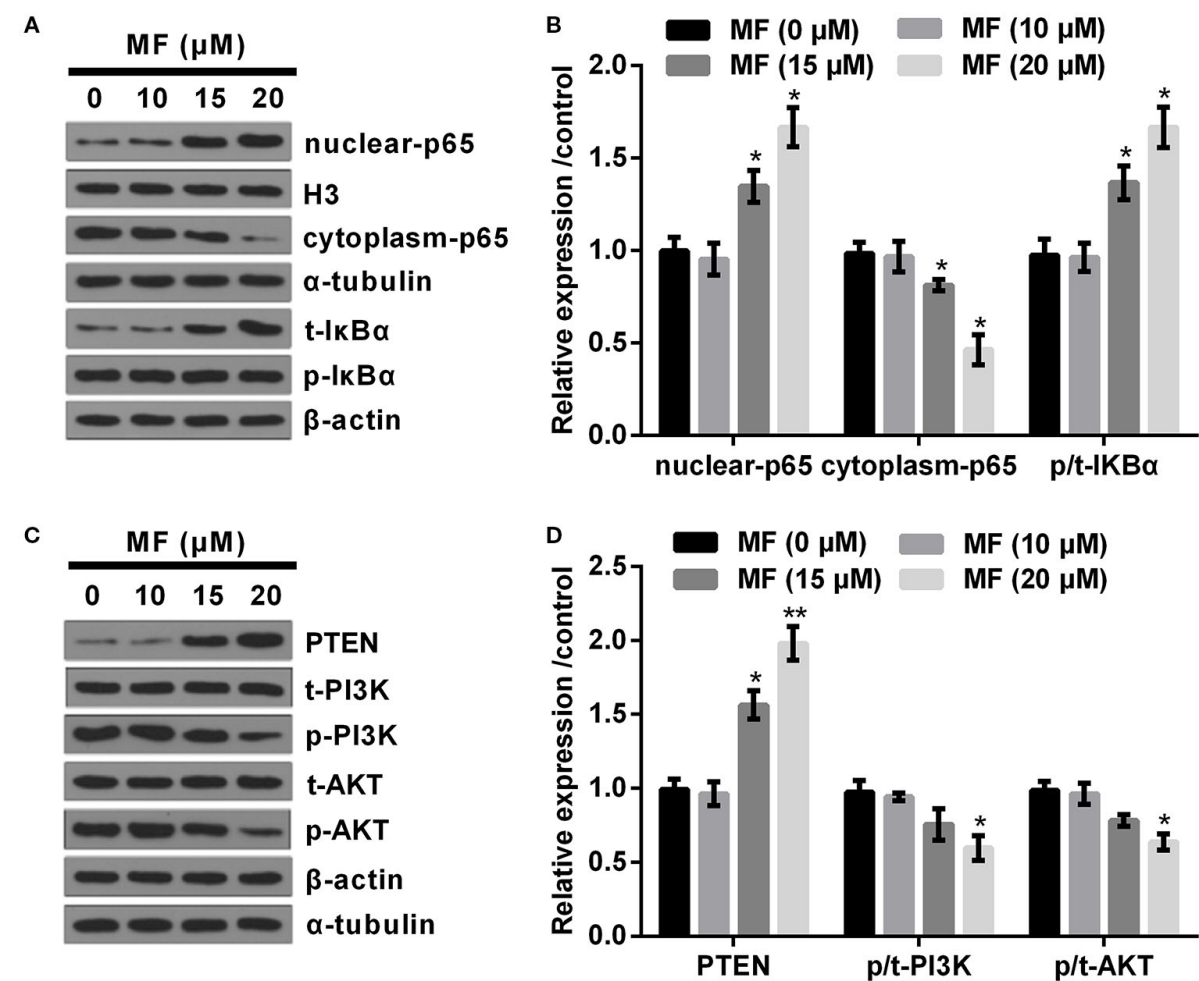
High doses of MF treatment activated NF-κB pathway, while deactivated PTEN/PI3K/AKT pathway. ATDC5 cells were exposed to series concentrations of MF (10, 15, and 20 μM). **(A**, **B)** 15 and 20 μM of MF treatment notably increased the expression of nuclear-p65 and the ratio of p/t-IκBα, while declined the expression of cytoplasm-p65. **(C**, **D)** 15 and 20 μM of MF treatment markedly increased the expression of PTEN, while 20 μM of MF treatment noticeably declined the ratios of p/t-PI3K and p/t-AKT. MF, mangiferin; NF-κB, nuclear factor kappa B; PTEN/PI3K/AKT, phosphate and tension homology deleted on chromosome 10/phosphatidylinositol 3 kinase/protein kinase B; IκBα, nuclear factor of kappa light polypeptide gene enhancer in B-cells inhibitor, alpha. *P < 0.05, **P < 0.01.

Based on the above observations, we performed high concentrations of MF (10, 15, and 20 μM) treatment on ATDC5 cells to determine its influences on NF-κB and PTEN/PI3K/AKT pathways. Results showed that 10 μM of MF treatment had no significant influence on the expression of nuclear-p65, cytoplasm-p65, and the ratio of p/t-IκBα. However, the expression of nuclear-p65 and the ratio of p/t-IκBα were both remarkably increased (*P* < 0.05), while cytoplasm-p65 was markedly decreased (*P* < 0.05, **Figures 2A, B**) when the concentrations of MF increased to 15 and 20 μM. As for PTEN/PI3K/AKT pathway, the results displayed that 10 μM of MF treatment had no significant influence on the expression of PTEN and the ratios of p/t-PI3K and p/t-AKT. However, the expression of PTEN were notably enhanced at 15 and 20 μM of MF treatment (*P* < 0.05 or *P* < 0.01), while the ratios of p/t-PI3K and p/t-AKT were both declined at 20 μM of MF treatment (*P* < 0.05, **Figures 2C, D**). These outcomes indicated that the NF-κB pathway was distinctly activated, while the PTEN/PI3K/AKT pathway was notably deactivated by high concentrations of MF treatment.

### MF Treatment Attenuated LPS-Evoked Injury in ATDC5 Cells

**Figure 3:**
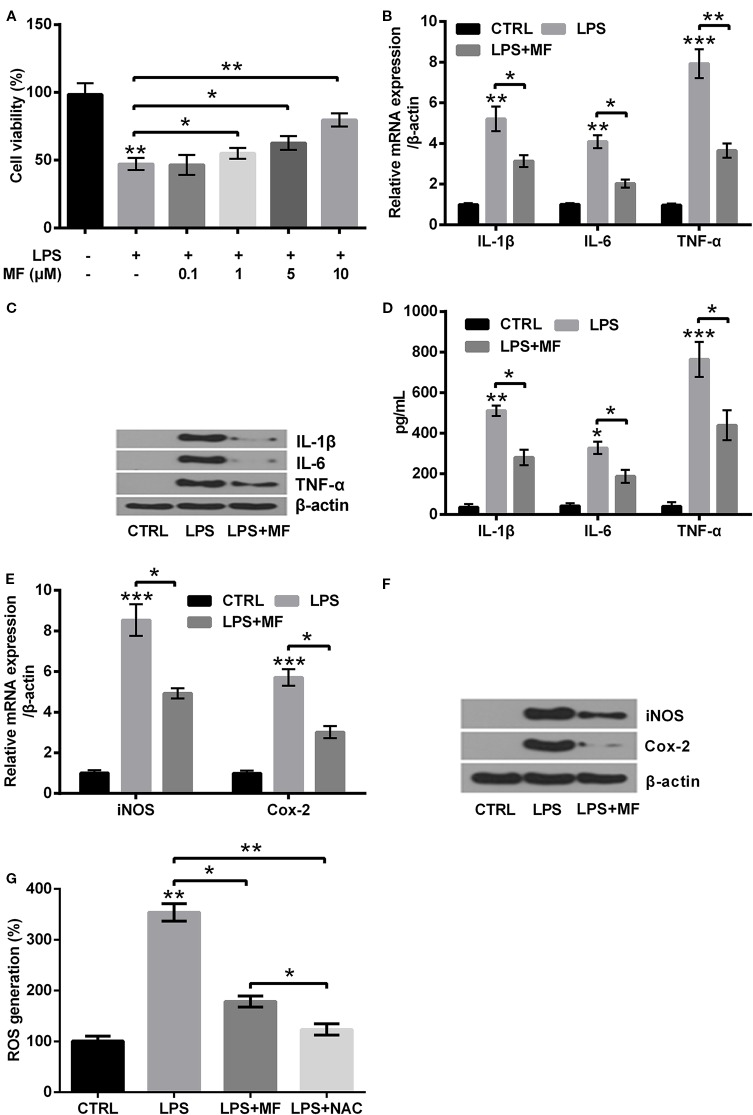
MF treatment attenuated LPS-evoked inflammatory injury in ATDC5 cells. **(A)** ATDC5 cells were pretreated with series concentrations of MF (0.1, 1, 5, 10, 15, and 20 μM) and induced with LPS. LPS inducement notably decreased the viability of ATDC5 cells. LPS inducement remarkably reduced cell viability, while MF exposure markedly increased cell viability and exhibited a dose-dependent manner. **(B**–**D)** LPS treatment markedly increased the messenger RNA (mRNA) and protein productions of pro-inflammatory cytokines (IL-6, IL-1β, and TNF-α) in ATDC5 cells, while MF exposure notably relieved the promoting effects. **(E**, **F)** LPS inducement markedly elevated the mRNA and protein expression of pro-inflammatory enzymes (iNOS and Cox-2) in ATDC5 cells, while MF exposure remarkably relieved the promoting effects. **(G)** LPS inducement markedly increased ROS generation in ATDC5 cells, while MF exposure exhibited a NAC-like effect and notably relieved LPS-triggered promoting effects. MF, mangiferin; LPS, lipopolysaccharide; IL, interleukin; TNF-α, tumor necrosis factor-α; Cox-2, cyclooxygenase-2; iNOS, inducible nitric oxide synthase; ROS, reactive oxygen species; CTRL, control. *P < 0.05, **P < 0.01, ***P < 0.001.

To specify the influences of MF treatment on LPS-evoked ATDC5 cells, we examined the variations of cell viabilities, productions of pro-inflammatory cytokines (IL-1β, IL-6, and TNF-α) and enzymes (iNOS and Cox-2). Besides, the variation of ROS generation was also analyzed. Data displayed in **Figure 3A** demonstrated that LPS treatment notably suppressed cell viability (*P* < 0.01), while MF exposure dramatically relieved the inhibitory effects (*P* < 0.05 or *P* < 0.01) and the results were presented in a dose-dependent manner compared with the LPS-induced group. Considering that the viability was increased most obviously when the concentration of MF was 10 μM, therefore this concentration was selected as the appropriate concentration for following experiments. Detection of expression levels of pro-inflammatory cytokines specified that LPS inducement remarkably enhanced the expression of IL-6 (*P* < 0.01), IL-1β (*P* < 0.01), and TNF-α (*P* < 0.001, **Figures 3B, C**) at both mRNA and protein levels. Besides, the protein concentrations of these cytokines in cell culture supernatant were also verified to be notably increased by LPS inducement (*P* < 0.05 or *P* < 0.01 or *P* < 0.001, **Figure 3D**). Further study demonstrated that the mRNA and protein expression of potent pro-inflammatory enzymes (iNOS and Cox-2) and ROS generation were also remarkably enhanced by LPS inducement (*P* < 0.01 or *P* < 0.001, **Figures 3E–G**). However, these effects were all markedly attenuated by MF exposure (*P* < 0.05 or *P* < 0.01, **Figures 3B–G**). The impact of MF exposure on ROS generation was confirmed by the outcome of NAC treatment (P < 0.01), which exhibiting that MF treatment performed a NAC-like effect in LPS-induced ATDC5 cells (*P* < 0.05, **Figure 3G**). These outcomes indicated that MF exposure remarkably relieved LPS-induced inflammatory injury exhibiting as decreasing the productions of pro-inflammatory cytokines, enzymes, and the generation of ROS.

### MF Inhibited NF-κB Pathway While Activated PTEN/PI3K/AKT Pathway in LPS-Induced ATDC5 Cells

**Figure 4:**
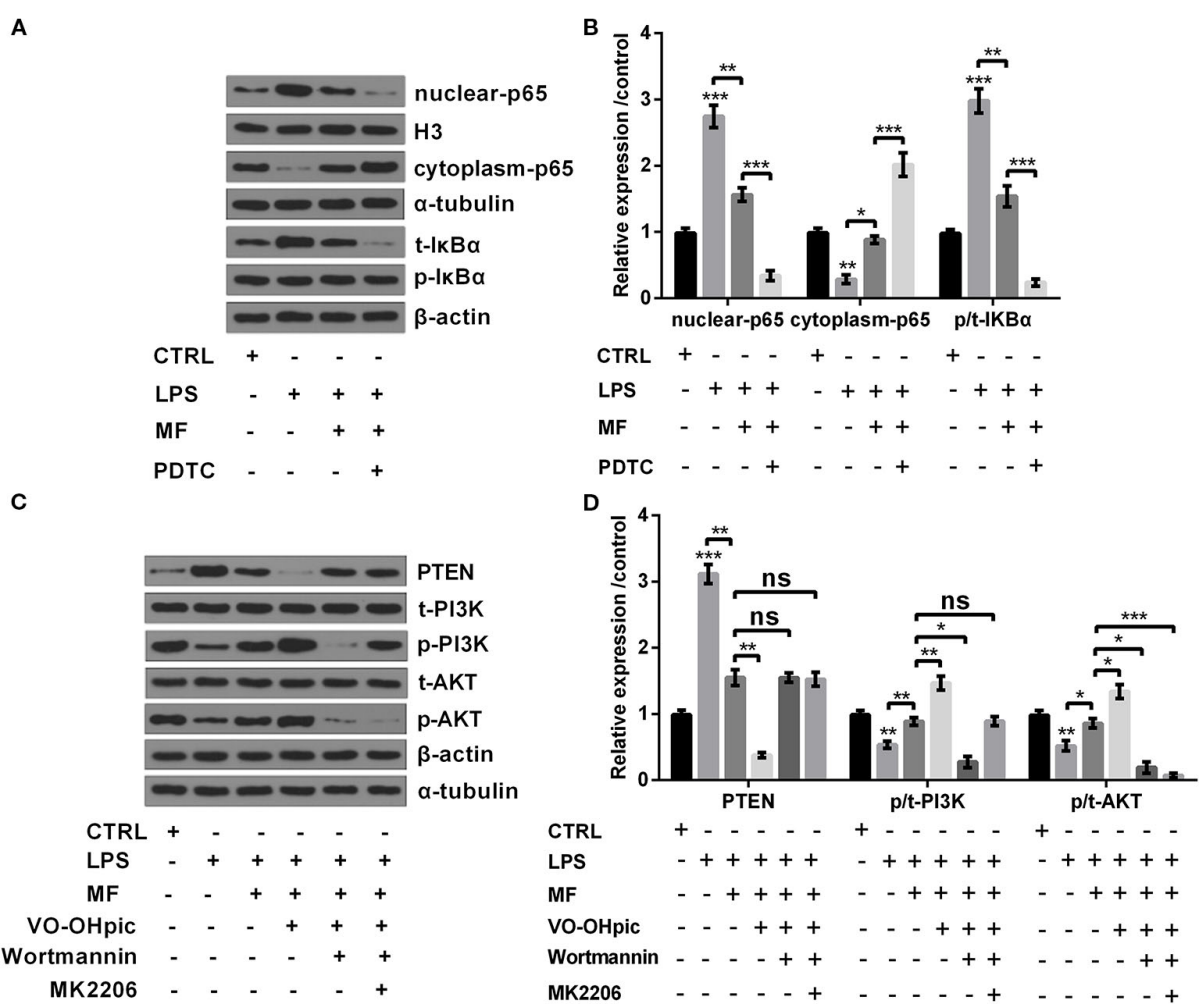
MF treatment inhibited NF-κB pathway while activated PTEN/PI3K/AKT pathway in LPS-induced ATDC5 cells. **(A**, **B)** MF treatment remarkably remitted LPS-induced promoting impacts on the expression of nuclear-p65 and the increased ratio of p/t-IκBα, as well as the inhibitory impact on the expression of cytoplasm-p65. However, the inhibitory impacts of MF treatment on NF-κB pathway were both blocked after addition of NF-κB pathway inhibitor pyrrolidine dithiocarbamate (PDTC). **(C**, **D)** MF treatment notably relieved LPS-triggered promoting impact on the expression of PTEN and the declining impacts on the ratios of p/t-PI3K and p/t-AKT. However, the promoting impacts of MF treatment on PTEN/PI3K/AKT pathway were blocked after addition of PTEN inhibitor VO-OHpic, PI3K inhibitor Wortmannin and AKT inhibitor MK2206. MF, mangiferin. LPS, lipopolysaccharide; NF-κB, nuclear factor kappa B; PTEN/PI3K/AKT, phosphate and tension homology deleted on chromosome 10/phosphatidylinositol 3 kinase/protein kinase B; IκBα, nuclear factor of kappa light polypeptide gene enhancer in B-cells inhibitor, alpha, ns, no significance. *P < 0.05, **P < 0.01, ***P < 0.001.

In addition to cell viability, productions of pro-inflammatory cytokines, enzymes and ROS generation, the influences of MF treatment on NF-κB and PTEN/PI3K/AKT pathways were also determined. Results showed that LPS inducement remarkably increased the expression of NF-κB pathway-related nuclear-p65 and the ratio of p/t-IκBα (both *P* < 0.001), while notably decreased the expression of cytoplasm-p65 (*P* < 0.01, **Figures 4A, B**). As for PTEN/PI3K/AKT pathway, results showed that LPS inducement dramatically promoted the expression of PTEN (*P* < 0.001), while notably declined the ratios of p/t-PI3K and p/t-AKT (both *P* < 0.01, **Figures 4C, D**). However, following trials disclosed that MF treatment distinctly relieved LPS-induced impacts on NF-κB and PTEN/PI3K/AKT pathways (*P* < 0.05 or *P* < 0.01, **Figures 4A–D**). In addition, further experiments disclosed that the expression of nuclear-p65 and the ratio of p/t-IκBα were both distinctly declined, while the expression of cytoplasm-p65 was markedly up-graded after addition of NF-κB pathway inhibitor PDTC (all *P* < 0.001, **Figures 4A, B**). Moreover, the expression of PTEN was remarkably declined, while the ratios of p/t-PI3K and p/t-AKT were both notably increased after addition of PTEN inhibitor VO-OHpic (*P* < 0.05 or *P* < 0.01). Besides, PI3K inhibitor Wortmannin treatment remarkably reduced the ratios of p/t-PI3K and p/t-AKT, while had no significant influence on the expression of PTEN (both *P* < 0.05). Additionally, addition of AKT inhibitor MK2206 notably decreased the ratio of p/t-AKT, while had no remarkably influence on the expression of PTEN and the ratio of p/t-PI3K (*P* < 0.001, **Figures 4C, D**). Collectively, these observations further confirmed that MF preformed its protective roles through regulation of NF-κB and PTEN/PI3K/AKT pathways.

### MF Enhanced the Expression of miR-181a

**Figure 5:**
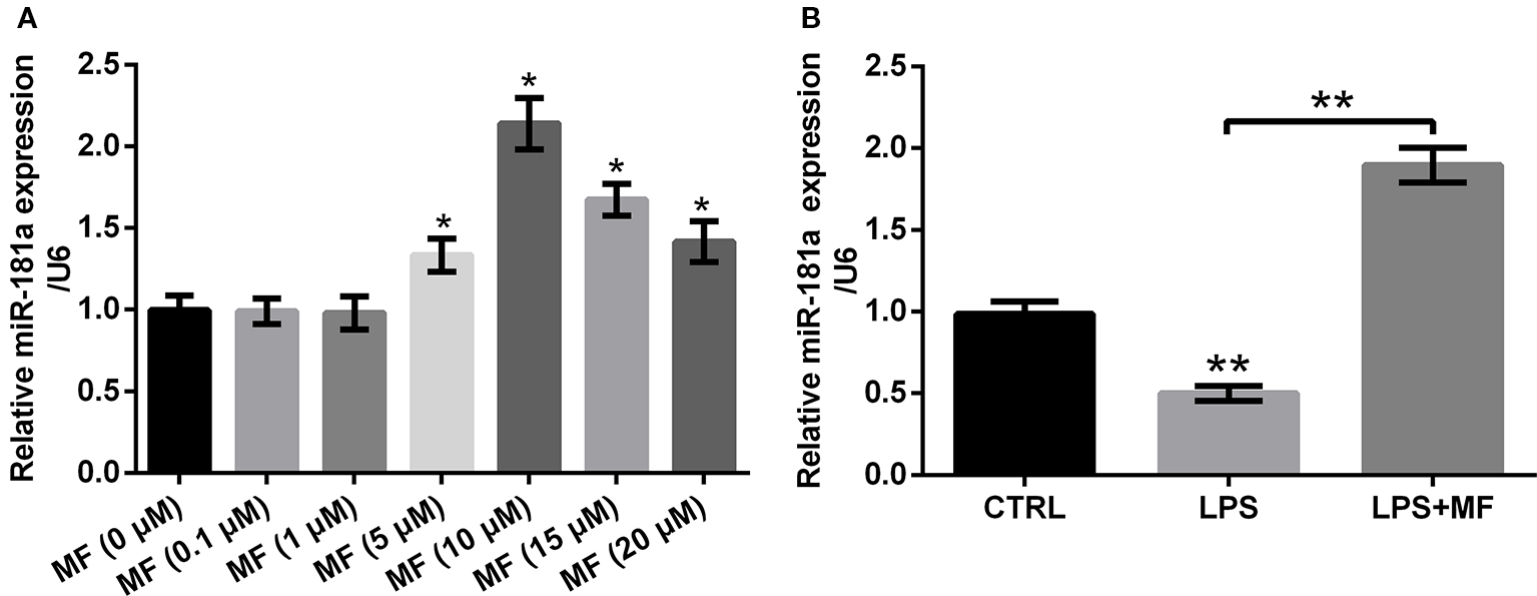
MF treatment enhanced miR-181a expression. ATDC5 cells were respectively treated with series concentrations of MF. ATDC5 cells were pre-treated with MF and then were induced with LPS. **(A)** The expression of miR-181a was markedly increased with the increased concentration of MF (5, 10, 15, and 20 μM). **(B)** LPS inducement markedly repressed miR-181a expression, while MF exposure dramatically reversed the repressive effects on miR-181a expression. MF, mangiferin; miR-181a, microRNA-181a; LPS, lipopolysaccharide; CTRL, control. *P < 0.05, **P < 0.01.

To specify the influences of series concentrations of MF treatment on miR-181a expression, we tested miR-181a expression by utilizing qRT-PCR. The results illustrated that 0.1 and 1 μM of MF treatment had no noticeable influence on the expression of miR-181a, while miR-181a expression was notably augmented with the increased concentration of MF (5, 10, 15 and 20 μM) (*P* < 0.05, **Figure 5A**). Further trials disclosed that LPS treatment significantly reduced miR-181a expression (*P* < 0.01), while this inhibitory impact was totally reversed by MF treatment (*P* < 0.01, **Figure 5B**). These results manifested that miR-181a might have participated in the function of MF in LPS-induced ADTC5 cells.

### PTEN was a Target of miR-181a

**Figure 6:**
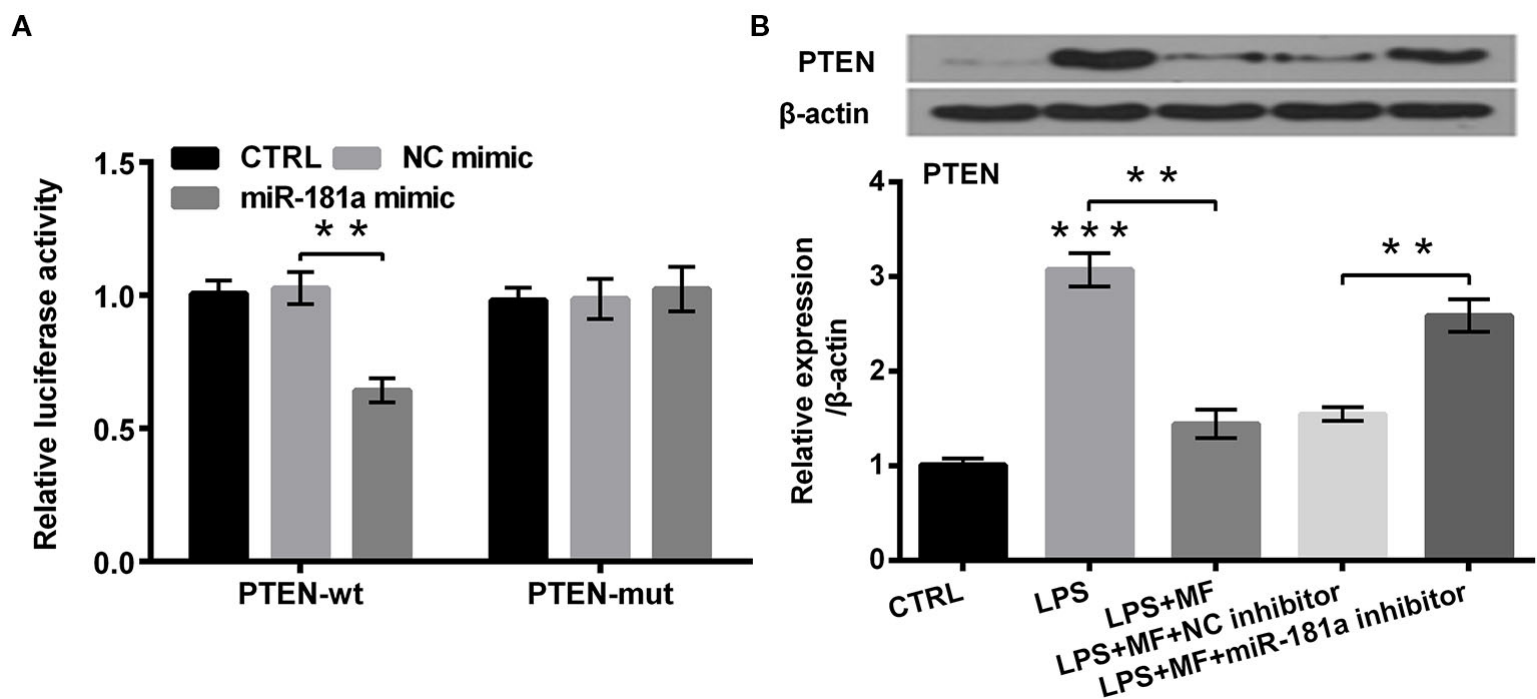
PTEN was a target of miR-181a. ATDC5 cells were respectively co-transfected with the pMiR-report vector PTEN-wt/PTEN-mut and the sequences of miR-181a mimic/NC mimic. ATDC5 cells were respectively transfected with miR-181a inhibitor and NC inhibitor, and then were treated with MF and LPS. **(A)** The luciferase activity of PTEN-wt and miR-181a mimic co-transfected group was noticeably decreased, while there was no significant influence on the luciferase activity of the PTEN-mut and NC mimic co-transfected group. These results meant that PTEN was a target of miR-181a. **(B)** LPS inducement notably enhanced PTEN expression, while MF treatment remarkably relieved this impact. Additionally, miR-181a inhibition markedly abolished MF-triggered impacts on PTEN expression. PTEN, phosphate, and tension homology deleted on chromosome 10. miR-181a, microRNA-181a; wt, wild type; mut, mutant type; NC, negative control; MF, mangiferin; LPS, lipopolysaccharide; CTRL, control. **P < 0.01, ***P < 0.001.

To explore how miR-181a was involved in the process of MF attenuated LPS-induced cell damage, we analyzed the probable target gene of miR-181a. The outcomes showed that co-transfection of PTEN-wt and miR-181a mimic dramatically reduced the luciferase activity of PTEN-wt group (*P* < 0.01, **Figure 6A**), while co-transfection of PTEN-mut and miR-181a mimic had no significant influence on the luciferase activity. Besides, western blot outcomes demonstrated that PTEN expression was markedly increased by LPS inducement (*P* < 0.001), while MF treatment dramatically remitted this effect (*P* < 0.01). Moreover, miR-181a inhibition partially reversed MF-triggered effects in LPS + MF induced ATDC5 cells (*P* < 0.01, **Figure 6B**). Combination of these outcomes led a conclusion demonstrating that PTEN was a target gene of miR-181a.

### MF Attenuated LPS-Induced Cellular Inflammatory Damage by Up-Regulating miR-181a Expression

**Figure 7:**
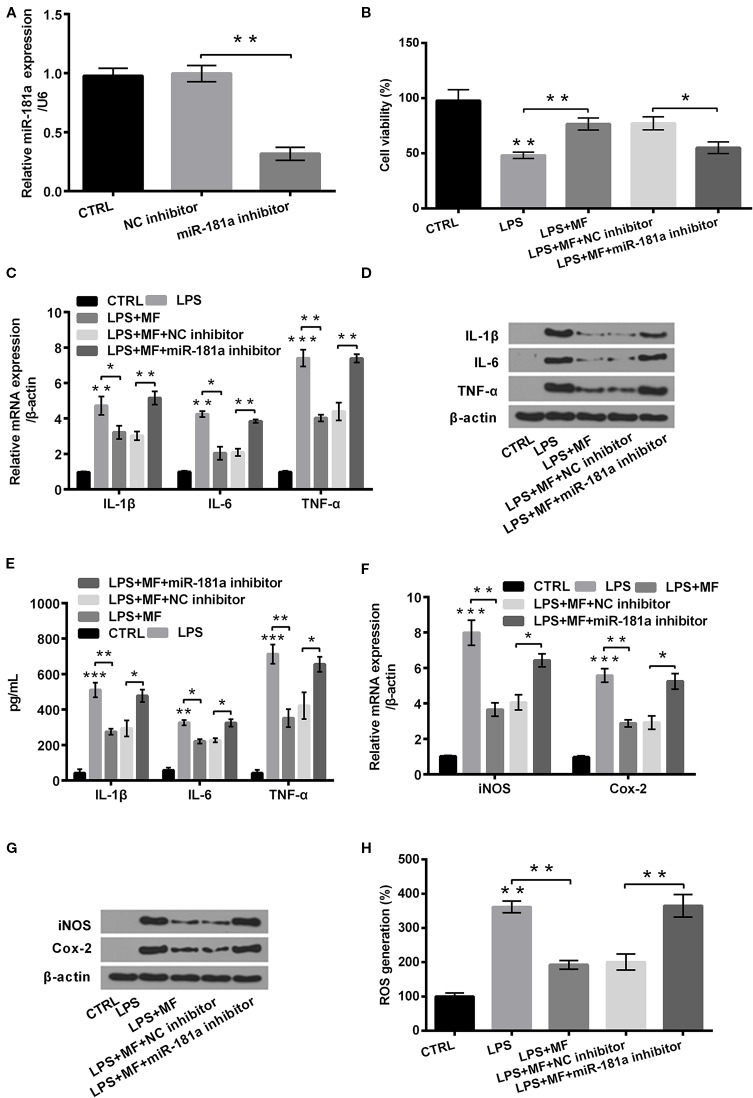
MF treatment attenuated LPS-induced cellular inflammatory damage by up-regulating the expression of miR-181a. ATDC5 cells were respectively transfected with miR-181a inhibitor and negative control (NC) inhibitor, and then were respectively treated with MF and LPS. **(A)** miR-181a expression was dramatically repressed after transfection with miR-181a inhibitor. **(B)** miR-181a inhibition remarkably reversed MF-evoked promoting effects on the viability of LPS-evoked ATDC5 cells. **(C**–**E)** miR-181a inhibition markedly reversed MF-evoked inhibitory effects on messenger RNA (mRNA) and protein productions of pro-inflammatory cytokines [interleukin (IL)-6, IL-1β, and TNF-α] in LPS-induced ATDC5 cells. **(F**, **G)** miR-181a inhibition markedly reversed MF-evoked inhibitory effects on messenger RNA (mRNA) and protein productions of pro-inflammatory enzymes (iNOS and Cox-2) in LPS-induced ATDC5 cells. **(H)** miR-181a inhibition markedly reversed MF-evoked inhibitory effects on ROS generation in LPS-induced ATDC5 cells. MF, mangiferin; LPS, lipopolysaccharide; IL, interleukin; TNF-α, tumor necrosis factor-α; Cox-2, cyclooxygenase-2; iNOS, inducible nitric oxide synthase; ROS, reactive oxygen species; miR-181a, microRNA-181a; CTRL, control. *P < 0.05, **P < 0.01, ***P < 0.001.

To uncover how miR-181a was involved in the regulating process of MF alleviated LPS-induced cell damage, we inhibited miR-181a expression in LPS + MF exposed ATDC5 cells. Data displayed in **Figure 7A** verified that miR-181a was notably inhibited in miR-181a inhibitor transfected ATDC5 cells (*P* < 0.01). Outcomes displayed in **Figure 7B** demonstrated that miR-181a inhibition notably reversed MF-triggered promoting effects on the viability of LPS + MF induced ATDC5 cells (*P* < 0.05). Besides, miR-181a inhibition also markedly counteracted the inhibitory effects that MF triggered on the expression of pro-inflammatory cytokines (IL-1β, IL-6, and TNF-α) at both mRNA and protein levels (all *P* < 0.01, **Figures 7C, D**). Moreover, the expression of these proteins in cell culture supernatant exhibited the same variation trends after miR-181a inhibition (all *P* < 0.05, **Figure 7E**). Furthermore, detection outcomes of the expression of potent pro-inflammatory enzymes (iNOS and Cox-2) also illustrated that miR-181a inhibition effectively abolished the suppressive effects that MF triggered in LPS + MF exposed cells ATDC5 (both *P* < 0.05, **Figures 7F, G**). In addition to this, ROS assay verified that miR-181a inhibition significantly counteracted MF-triggered inhibitory impact on ROS generation (*P* < 0.01, **Figure 7H**). These results illustrated that MF might have attenuated LPS-induced inflammatory damage by increasing miR-181a expression.

### MF Inhibited NF-κB Pathway While Activated PTEN/PI3K/AKT Pathway Through Up-Regulating miR-181a Expression

**Figure 8:**
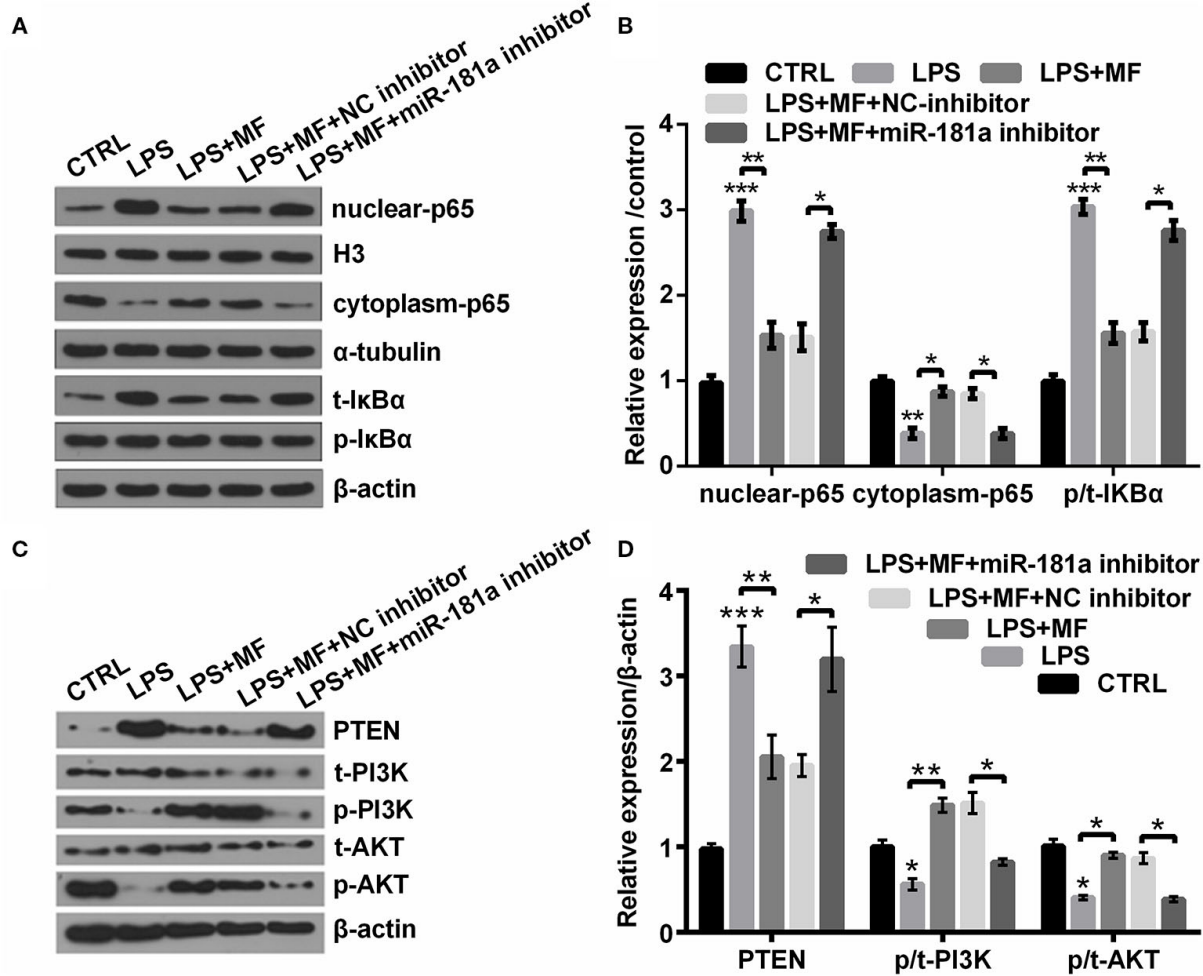
MF inhibited NF-κB pathway while activated PTEN/PI3K/AKT pathway through up-regulation of miR-181a. ATDC5 cells were respectively transfected with miR-181a inhibitor and NC inhibitor, and then were respectively treated with MF and LPS. **(A**, **B)** miR-181a inhibition partially counteracted MF-evoked repressive effects on the expression of nuclear-p65 and the ratio of p/t-IκBα, as well as the promoting effect on the expression of cytoplasm-p65 in LPS-induced ATDC5 cells. **(C**, **D)** miR-181a inhibition partially counteracted MF-evoked repressive effects on the expression of PTEN and the promoting effects on the ratios of p/t-PI3K and p/t-AKT in LPS-induced ATDC5 cells. MF, mangiferin; LPS, lipopolysaccharide; miR-181a, microRNA-181a; NC, negative control; NF-κB, nuclear factor kappa B; PTEN/PI3K/AKT, phosphate and tension homology deleted on chromosome 10/phosphatidylinositol 3 kinase/Protein kinase B; IκBα, nuclear factor of kappa light polypeptide gene enhancer in B-cells inhibitor, alpha. *P < 0.05, **P < 0.01, ***P < 0.001.

To probe into the underlying mechanism of MF relieved LPS-induced cell damage, we investigated the expression of vital factors participated in NF-κB and PTEN/PI3K/AKT pathways. Outcomes displayed in **Figures 8A, B** suggested that LPS inducement notably enhanced the expression of nuclear-p65 and increased the ratio of p/t-IκBα (both *P* < 0.001), while declined the expression of cytoplasm-p65 (*P* < 0.01). Results displayed in **Figures 8C, D** suggested that LPS inducement notably elevated the expression of PTEN (*P* < 0.001), while markedly decreased the ratios of p/t-PI3K and p/t-AKT (both *P* < 0.05). However, following results indicated that MF exposure remarkably remitted LPS-induced impacts (*P* < 0.05 or *P* < 0.01, **Figures 8A–D**). Besides, miR-181a inhibition partially reversed MF-triggered impacts on LPS-induced ATDC5 cells (all *P* < 0.05, **Figures 8A–D**). These outcomes manifested that MF may have inhibited NF-κB pathway and activated PTEN/PI3K/AKT pathway *via* increasing the expression of miR-181a.

## Discussion

In this investigation, high concentrations of MF treatment (15 and 20 μM) effectively induced inflammatory injury in ATDC5 cells exhibiting as remarkably increased the productions of pro-inflammatory cytokines, enzymes, and generation of ROS. While 10 μM of MF treatment notably relieved LPS-induced inflammatory injury, deactivated NF-κB pathway and activated PTEN/PI3K/AKT pathway. PTEN was verified to be a target of miR-181a. Inhibition of miR-181a markedly counteracted MF-triggered effects in LPS-induced ATDC5 cells. These outcomes indicated that MF might play its protective roles in LPS-induced ATDC5 cells through increasing the expression of miR-181a.

It was reported that LPS could contribute to low-grade cell inflammation (Huang and Kraus, 2016), while OA is exactly the low-grade inflammatory state mentioned above. Besides, previous investigations have demonstrated that MF was a xanthonoid with anti-inflammatory capacity, and it was reported to be a promising therapeutic drug for OA (Luczkiewicz et al., 2014; Saha et al., 2016; Qu et al., 2017b; Garrido-Suarez and Garrido, 2019). Previous investigations elucidated that MF markedly suppressed the productions of IL-6, IL-1β, and TNF-α in LPS-triggered primary hepatocytes, BALB/c mice mammary gland, and peritoneal macrophages (Jeong et al., 2014; Pan et al., 2016; Qu et al., 2017a). Furthermore, an earlier research revealed that PT-MF remarkably decreased the expression of Cox-2, iNOS and TNF-α, and the generation of ROS in LPS-evoked RAW 264.7 cells (Bulugonda et al., 2017). In this investigation, we firstly explored the influences of MF treatment alone on the ATDC5 cells. Then, the impacts of MF on LPS-induced ATDC5 cells were investigated. Results showed that high concentrations of MF treatment alone remarkably induced inflammatory injury in ATDC5 cells, which meant that low concentrations of MF treatment might perform protective efficacy on ATDC cells, however excessive concentrations of MF treatment could have deteriorated the inflammatory injury. Besides, following experiments showed that MF exposure partially reversed LPS-induced impacts, exhibiting as increasing cell viability and down-regulating the productions of pro-inflammatory cytokines, enzymes, and ROS generation. These outcomes were consistent with earlier studies and our predictions, which hinted that MF treatment effectively alleviated LPS-induced inflammatory injury in ATDC5 cells.

Previous investigation reported that miRNAs participated in the process of OA pathogenesis (Goldring and Marcu, 2012). Besides, miR-181a was a member of the miRNAs mentioned above (Okuhara et al., 2012; Zhai et al., 2017). Thus, to clarify how MF participated in this regulatory process of MF in OA, we firstly examined miR-181a expression in MF exposed ATDC5 cells. The results illustrated that MF exposure dramatically elevated the level of miR-181a expression. Afterwards, miR-181a inhibition was conducted in LPS + MF treated ATDC5 cells to figure out how miR-181a functioned in the process of MF alleviated LPS-induced inflammatory injury. The results turned out to be that miR-181a inhibition diminished MF-triggered promoting effects on cell viability, and repressing effects on the expression of pro-inflammatory cytokines, enzymes, and ROS generation. These observations indicated that miR-181a might perform anti-inflammatory efficacy in LPS-induced ATDC5 cells, which was in line with earlier reports (Xu et al., 2015b; Du and Lu, 2018; Jiang et al., 2018). Combination of our results and previous investigation led to a conclusion demonstrating that MF may have relieved LPS-evoked damaged through up-regulating miR-181a expression.

Previously studies specified that numbers of miRNAs functioned in OA through targeting PTEN. It was demonstrated that miR-181 restrained cell proliferation and promoted cell apoptosis through targeting PTEN in OA (Wu et al., 2017b). Besides, a previous investigation clarified that miR-337-3p enhanced proliferation while suppressed cell apoptosis *via* targeting PTEN/AKT axis in OA either (Huang et al., 2017). Another research revealed that miR-130a alleviated OA by targeting PTEN (Zhang et al., 2018). Hence, to clarify how miR-181a functioned in the progress of MF alleviating LPS-induced injury, we explored the target gene of miR-181a. Following results demonstrated that PTEN was a target of miR-181a, which was in line with earlier studies aforementioned. Thus, these outcomes hinted that MF could have relived LPS-induced inflammatory injury through regulating miR-181a/PTEN axis.

Nowadays, accumulating studies have demonstrated that MF exerted its regulatory roles through regulating NF-κB and PTEN/PI3K/AKT pathways. A previous study revealed that suppression of NF-κB could effectively alleviate OA development (Roman-Blas and Jimenez, 2006). Besides, researchers have clarified that MF exposure could repress LPS-induced phosphorylation of NF-κB (Jeong et al., 2014; Tsubaki et al., 2015; Qu et al., 2017a). Earlier investigations demonstrated that the un-stimulated NF-κB was localized in the cytoplasm (Chen et al., 1999), while once stimulated NF-κB would be translocated into the nuclear and then activated its mediated transcription gene expression (Feng et al., 2012). Moreover, previous research verified that loganin treatment relieved IL-1β-induced ECM catabolism and apoptosis through regulating PI3K/AKT pathway in rat chondrocytes (Yang et al., 2019). Additionally, it was disclosed that miR-181 performed protective roles in OA through targeting PTEN (Wu et al., 2017b). Therefore, we examined the expression of vital proteins participated in NF-κB and PTEN/PI3K/AKT pathways for probing into the underlying mechanism of MF and miR-181a in the protective effects of LPS-induced ATDC5 cells in this investigation. The outcomes revealed that MF exposure dramatically diminished LPS-induced increased expression of nuclear-p65 and the ratio of p/t-IκBα, as well as LPS-induced decreased expression of cytoplasm-p65 that related to NF-κB pathway. Furthermore, MF exposure notably decreased LPS-induced enhanced expression of PTEN, while remarkably enhanced LPS-induced decreased ratios of p/t-PI3K and p/t-AKT. However, these impacts were all noticeably reversed by inhibition of miR-181a. These results were in line with earlier investigations and hinted that MF performed its roles in LPS-induced ATDC5 cells *via* deactivation of NF-κB pathway and activation of PTEN/PI3K/AKT pathway through elevating miR-181a expression.

## Conclusion

OA is a complex and common clinical disease, which is the result of the interaction of various factors, and its pathogenesis is complex, which is still not clear until now. Therefore, it’s essential to choose a reasonable method for OA modeling to investigate the pathogenesis. In this investigation, only one kind of cells was used for LPS stimulation, which was relatively simple and could not fully reflect the pathophysiological characteristics of OA. Besides, the results of our research disclosed that MF was highly effective in protecting ATDC5 cells against LPS-induced inflammatory injury, while other researches about the efficacy of scutellarin and loganin were largely focused on their functions on cartilage degeneration (Hu et al., 2020; Liu et al., 2020). Therefore, further researches still need to be done using multi-cells, multi-stimulation and animal models to disclose the pathogenesis of OA, and identifying the most effective therapeutic strategies for the treatment of OA.

Overall, this investigation manifested that MF treatment attenuated LPS-induced cellular inflammatory damage exhibiting as increasing the viability and declining the productions of inflammatory-cytokines and enzyme, as well as the generation of ROS *via* deactivation of NF-κB pathway and activation of PTEN/PI3K/AKT pathway through regulation of miR-181a.

## Data Availability Statement

The raw data supporting the conclusions of this article will be made available by the authors, without undue reservation, to any qualified researcher.

## Author Contributions

Conception and design: YunfM, YL, YunyM, NJ, QL, and BY. Administrative support: YunfM, LW, QL, and BY. Provision of study materials or patients: YunfM, LW, YunyM, WN, QL, and BY. Collection and assembly of data: YunfM, YL, YunyM, BW, and YH. Data analysis and interpretation: YunfM, LW, YH, and NJ. Manuscript writing: All authors. Final approval of manuscript: All authors.

## Funding

The study was supported by grants from the China Postdoctoral Science Foundation (2018M633075).

## Conflict of Interest

The authors declare that the research was conducted in the absence of any commercial or financial relationships that could be construed as a potential conflict of interest.

## Abbreviations

ANOVA, analysis of variance; CCK-8, Cell Counting Kit-8; DPBS, Dulbecco's Phosphate Buffered Saline; ELISA, Enzyme-linked immunosorbent assay; FBS, fetal bovine serum; IL, interleukin; LPS, lipopolysaccharide; MF, Mangiferin; miRNAs, microRNAs; MUT, mutant; NF-κB, nuclear factor kappa; OA, Osteoarthritis; qRT-PCR, Quantitative real-time polymerase chain reaction; ROS, oxygen species; SD, standard deviation; TBST, Tris-Buffered Saline Tween; WT, wild type.
